# Turner Syndrome and Hepatic Adenomas: A Case Report

**DOI:** 10.7759/cureus.96137

**Published:** 2025-11-05

**Authors:** Susan Ahern, Sindy H Wei

**Affiliations:** 1 Endocrinology, Diabetes and Metabolism, University of California, Los Angeles, USA; 2 Abdominal Imaging, University of California, Los Angeles, USA

**Keywords:** hepatic adenoma, large hepatic adenomas on oral combined contraceptive, metabolic syndrome in turner syndrome, transaminase elevation in turner syndrome, turner syndrome

## Abstract

This case report describes large hepatic adenomas in a 35-year-old woman with Turner syndrome (TS) who had long-standing oral estradiol exposure and metabolic syndrome. Patients with TS may be at increased risk of hepatic adenomas when compared to the general population. Future research papers should seek to understand the true incidence of hepatic adenomas in TS, whether we should implement routine imaging screening for hepatic adenomas without abnormal liver function labs, and whether transdermal estradiol is safe after a diagnosis of hepatic adenomas.

## Introduction

Hepatic adenomas are relatively rare, with an estimated annual incidence in the general population of 1 per 1,000,000 [1,2]. The prevalence is estimated to be 0.04% in the general population; however, the true prevalence is unclear as hepatic adenomas are often asymptomatic and can resolve spontaneously [1,3]. Based on data from a few small studies and case reports in the literature, the prevalence of hepatic adenomas may be as high as 30 times higher in Turner syndrome (TS) than in the general population [1,2,4]. Estrogen replacement therapy is associated with an increased risk of hepatic adenomas [1-4]. Estrogen replacement therapy is a mainstay of treatment in TS [1,5]. Patients with TS also have an increased risk of hepatic steatosis and cirrhosis [1,5-7]. Hepatic adenomas are diagnosed from liver imaging [2,3]. Some but not all patients may have serum liver function abnormalities [1,5]. Hepatic adenomas can have complications, including rupture, and may have malignant potential [1-4]. Our case contributes to the literature on hepatic adenomas in patients with TS and supports the currently recommended ultrasound screening in those with abnormal liver enzymes [5]. It also highlights several knowledge gaps, specifically whether the prevalence of hepatic adenomas is higher in patients with TS than in the general population with the same exposure to oral estrogen, whether screening should be performed in those without liver enzyme elevations after a certain length of time on oral estrogen-containing contraceptives, and also about the safety of using transdermal estrogen once oral estrogen-containing contraceptives are discontinued in those with TS. 

## Case presentation

**Figure 1:**
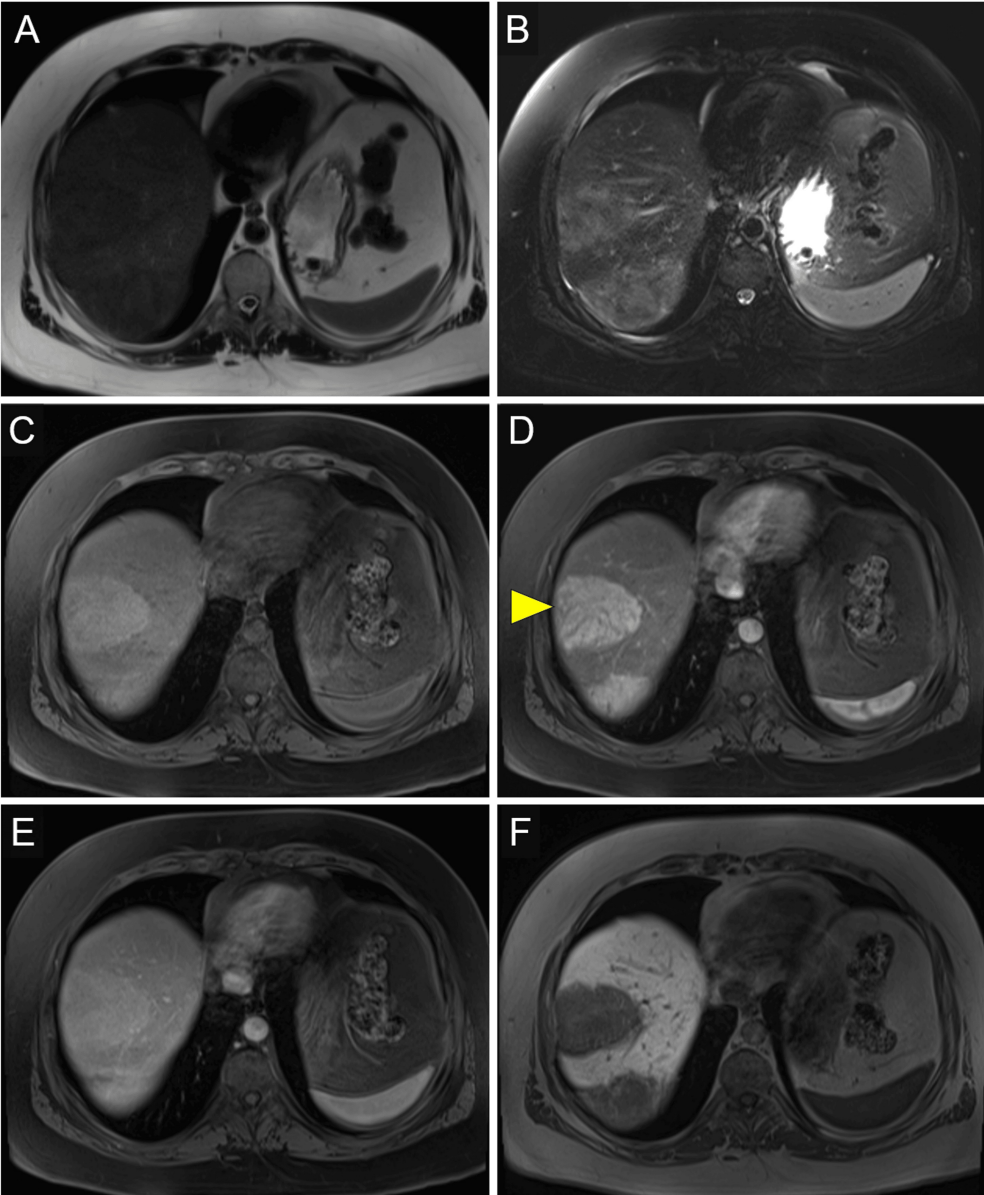
Hepatic adenoma segment VIII Hepatic adenoma in segment VIII measuring 6.7 x 5.5 cm (arrowhead). MRI sequences include T2-weighted (A), T2-weighted with fat saturation (B), and T1-weighted images with fat saturation obtained over multiple phases, including pre-contrast (C), post-contrast arterial phase (D), venous phase (E), and hepatobiliary phase (F).

**Figure 2:**
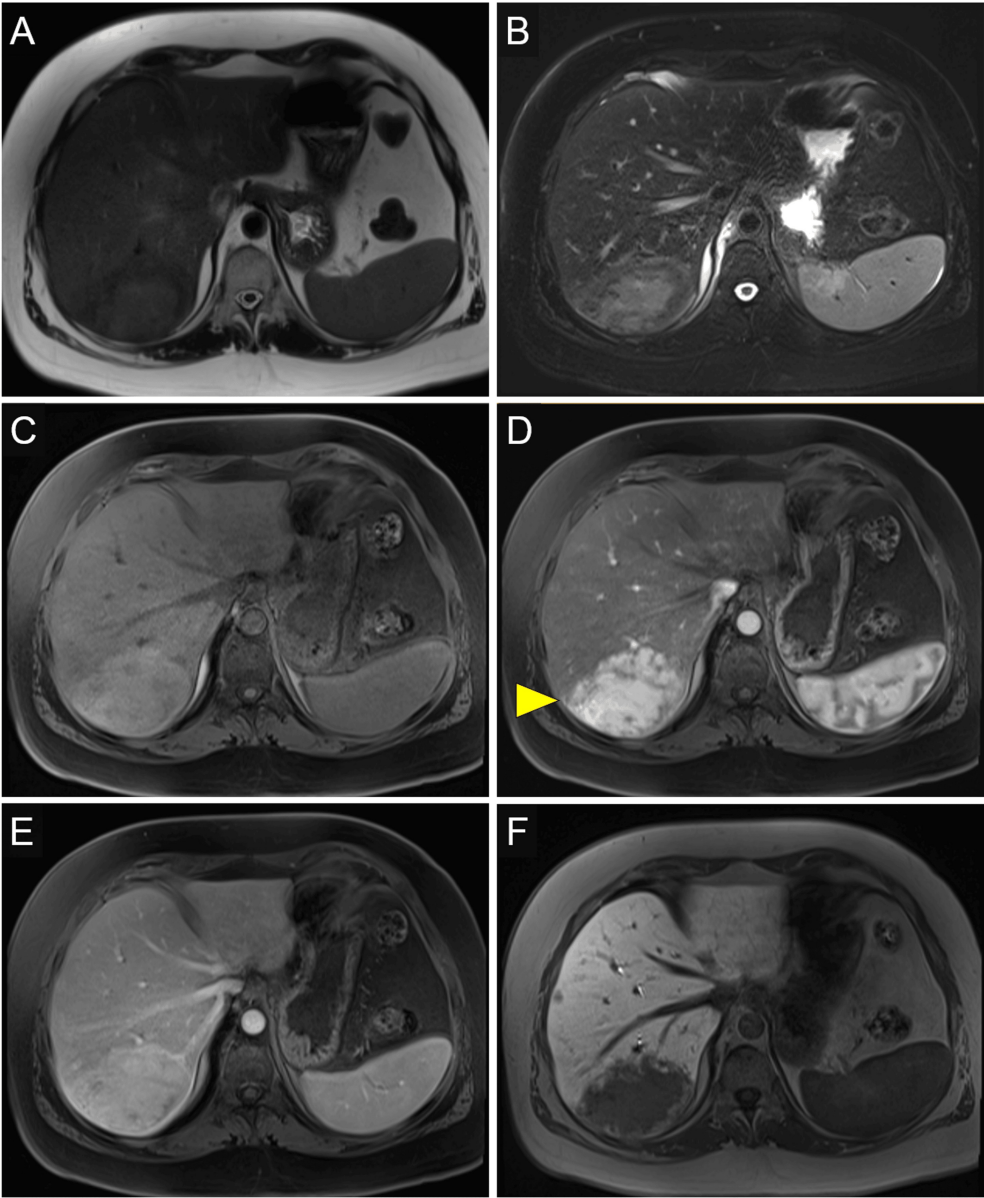
Hepatic adenoma in segment VI/VII Hepatic adenoma in segment VI/VII measuring 9.0 x 5.8 cm (arrowhead). MRI sequences include T2-weighted (A), T2-weighted with fat saturation (B), and T1-weighted images with fat saturation obtained over multiple phases, including pre-contrast (C), post-contrast arterial phase (D), venous phase (E), and hepatobiliary phase (F).

A 35-year-old woman presented to an adult endocrinology clinic for continued management of her TS and type 2 diabetes. She was originally diagnosed with TS at age 9 and was started on growth hormone therapy shortly after her diagnosis, followed by estrogen replacement four years later, around the age of 13. On review of the patient’s records, abnormal liver enzymes were noted at the time of her diagnosis at 9 years of age. At the time of her diagnosis, alanine aminotransferase and gamma-glutamyl transpeptidase were mildly elevated. In our adult endocrinology clinic, her presenting BMI was 33.1. With comprehensive lifestyle change and diabetes medical management, including the use of a glucagon-like peptide-1 receptor agonist, her BMI reduced to 30.9. Despite weight loss and clinical improvement in metabolic disease, her liver enzymes increased (Table 1). A subsequent liver ultrasound showed two large hypoechoic liver masses with mild hepatic steatosis and nodular liver surface. Liver MRI showed two large, mildly T2 hyperintense masses in the right lobe of the liver, measuring 6.7 x 5.5 cm, in liver segment 8 (Figures 1A-1F) and 9.0 x 5.8 cm, in liver segments 6 and 7 (Figures 2A-2F). Masses demonstrate arterial hyperenhancement, venous phase isointensity, and hepatobiliary phase hypointensity suggestive of hepatic adenomas.

**Table 1:** Serum hepatic function (H): Data is abnormally high. ALT: Alanine aminotransferase; AST: Aspartate aminotransferase; SGOT: Serum glutamic-oxaloacetic transaminase; SGPT: Serum glutamic-pyruvic transaminase.

	Latest reference range and units	06/19/2023 (10:01 AM)	09/20/2024 (06:12 PM)	12/19/2024 (08:10 PM)
AST (SGOT)	10-30 U/L	20	34 (H)	40 (H)
ALT (SGPT)	6-29 U/L	32 (H)	74 (H)	85 (H)
Alkaline phosphatase	31-125 U/L	338 (H)	314 (H)	331 (H)
Bilirubin, total	0.2-1.2 mg/dL	0.3	0.3	0.3
Albumin	3.6-5.1 g/dL	4.1	4.2	4.3
Albumin/globulin ratio (quest)	1.0-2.5 (calc)	1.6	1.8	2.2

Our patient consulted with endocrinology, hepatology, and hepatobiliary surgery and was presented at our institution’s tumor board. Given that an extensive right liver resection would be required, observation with serial imaging was chosen. Oral estradiol was discontinued. From a bone health perspective, her calcium and vitamin intake has been optimized. Three-month follow-up imaging showed a slight decrease in the size of adenomas.

## Discussion

TS is a genetic disorder caused by the complete or partial absence of an X chromosome, leading to short stature, neurocognitive difficulties, cardiovascular, renal, autoimmune, and metabolic diseases, as well as bone mineral disorders and hypogonadism [1,5]. TS affects approximately 1 in 2000-2500 phenotypic females [6,7-9]. Estrogen replacement is the mainstay of management in TS due to the associated ovarian insufficiency and hypergonadotropic hypogonadism. Estrogen replacement is important for breast development, bone health, and cardiometabolic health in TS. Guidelines written by Gravholt et al. suggest starting estrogen around the age of 11-12 years if follicle-stimulating hormone is elevated twice on separate measurements and gradually increasing to adult replacement over 2-4 years. Transdermal estrogen replacement is preferred to oral estradiol; however, oral is superior to not initiating estrogen replacement at all. Estrogen and progesterone treatment is recommended until the age of 50-55 years [5].

Hepatic steatosis and elevated transaminases are commonly observed in TS. Increased liver enzymes are present in 20%-80% of patients with TS [6,7]. The risk has been commonly attributed to obesity and estrogen exposure, but data suggest there may be other factors [9]. The risk of cirrhosis may be six times higher in TS than in the general population [1,5-7]. Guidelines suggest monitoring of hepatic function every 1-2 years starting at age 10. If persistent elevation of transaminases is present, then liver ultrasound and referral to a gastroenterologist are recommended [5,7]. The pathophysiology of hepatic damage may be steatosis, steatohepatitis, biliary disease, cirrhosis, vascular damage in regenerative nodular hyperplasia, and autoimmune disease [5,6]. Patients with TS have more visceral adiposity and metabolic-associated fatty liver disease. This is the most common cause of elevated liver enzymes in TS [5]. Currently, TS guidelines do not recognize patients with TS as being at a higher risk of hepatic adenomas.

Hepatic adenomas have been reported in TS and may occur at a rate that is higher than the general population [1,4]. Hepatic adenomas are rare epithelial tumors of the liver occurring at approximately one per million in the general population [1-3]. Individuals who use oral estrogen-containing contraceptives may have a fourfold increased risk [1-4]. Complications of hepatic adenomas can include hemorrhage and malignant transformation [1-4]. Hepatic imaging in TS is not currently routinely recommended. Management of hepatic adenoma may include elective resection for lesions over 5 cm [2,3].

A retrospective chart review study, looking at 228 patients with TS, showed that of the roughly one-half of patients who had liver function tests available to review, 48.6% were abnormal. About 77 patients had hepatic imaging performed, and 5 had abnormalities. Three patients, or 1.3%, had hepatic adenomas. One patient was diagnosed at one month old. A second was a 24-year-old woman who presented with hemorrhagic shock from rupture of a 9.5 cm hepatic adenoma. She had received at least two years of estrogen therapy before her diagnosis. The last case was of a 31-year-old who had a 0.9 cm hepatic adenoma by imaging, and this lesion increased over five years despite discontinuing exogenous estrogen. Interestingly, all three of these patients had normal liver chemistries [1]. This data suggests that hepatic adenoma may be more common in TS than in the general population. Routine monitoring of liver function is already recommended but may not always identify cases of hepatic adenomas.

There are only a few other case reports of hepatic adenomas in TS. One report from Nemoto et al. in 2019 reported a 36-year-old woman with TS who had been on oral contraceptive for 20 years and who had 6 cm and 1 cm hepatic adenomas. Her tumors were resected, and oral estrogen was stopped. There was no recurrence after 13 months [4]. Another case report describes a 13 year old with a hepatic adenoma who had been on growth hormone for three years [10]. There is also a case report detailing a patient with mosaic TS who had hepatic adenomas; however, information about the adenomas and estrogen exposure were not detailed and the author was not available for correspondence [11].

Hepatic adenomas have several proposed genetic abnormalities. Most hepatic adenomas, approximately 35%-40%, are from hepatic nuclear factor 1-alpha (HNF-1α) inactivated mutations. HNF-1α is involved in hepatocyte development, liver growth, and glucose and lipid metabolism [2,3]. A smaller portion of cases, approximately 15%-20%, are associated with β-catenin-activated mutations. These mutations are linked with androgen exposure, glycogenesis, and familial adenomatous polyposis. β-catenin mutations are not associated with steatosis or inflammatory conditions [2,3]. There is evidence that the Wnt/β-catenin pathway is dysregulated in TS. These adenomas may be particularly predisposed to malignant transformation [12]. Inflammatory hepatocellular adenomas are present in 40%-50% of cases with hepatic adenomas. Risk factors may be female, high BMI, excessive alcohol consumption, and systemic inflammation. These lesions do not have HNF-1⍺ or β-catenin mutations. The tumors show positivity for serum amyloid A and CRP [2,3]. 

Hepatic adenomas in TS may be from an inflammatory pathway, as there is a well-known increase in steatohepatitis in TS. There also may be risk associated with dysregulation of the β-catenin pathway. This may explain why there is at least one case report of very young individual with TS having a hepatic adenoma [2,3].

Use of any estrogen hormone replacement after the diagnosis of hepatic adenoma, even non-oral forms, is not recommended based on the clinical practice guideline written by the Association for the Study of Liver Diseases in 2021 [13]. There is a case report showing regression of hepatic tumors during transdermal hormone therapy [14]; however, this data in a population with TS is not available.

## Conclusions

Our case report and review of existing case reports highlight that patients with TS may be at increased risk for hepatic adenomas compared with the general population. The cases available in the literature vary significantly in the age of the patient, exposure to estrogen, and degree of serum liver function abnormalities. This suggests there are likely additional independent risk factors for hepatic adenoma in TS apart from estrogen exposure and hepatic steatosis; however, these factors remain unclear. Contrary to current guidelines, perhaps we should be using additional clinical criteria other than liver enzyme abnormalities as indications for imaging. As cases of hepatic adenomas in TS increase, perhaps guidelines should consider screening with ultrasound after a certain number of years on oral estrogen therapy, compared with routine screening at a specific age. It will be crucial to consider how to best optimize bone health in patients with TS after a hepatic adenoma is diagnosed, since it is currently accepted to stop all estrogen therapy, including non-oral forms, after diagnosis. It remains unclear if transdermal estradiol is safe to use after the discovery of hepatic adenoma. We need more information from pediatric populations to obtain incidence and prevalence data of hepatic adenomas and to determine safe monitoring practices and long-term estradiol management. 
